# Paternalistic prejudice in geopolitical perception: how low-competence stereotypes and cultural positivity jointly predict favorable evaluations of China–Africa relations

**DOI:** 10.3389/fpsyg.2026.1686945

**Published:** 2026-07-10

**Authors:** Yufang Ou, Man Ding, Qian Zhou

**Affiliations:** Institute of African Studies, College of African Area and Country Studies, Zhejiang Normal University, Jinhua, China

**Keywords:** China–Africa relations, cultural positivity, intergroup contact, paternalistic prejudice, stereotype content model

## Abstract

**Introduction:**

Chinese public perceptions of Africa remain understudied at the micro-level, yet they constitute a critical social-psychological foundation for the long-term sustainability of China-Africa bilateral relations. A central paradox motivates this research: how can negative stereotypes of Africa be positively associated with favorable evaluations of China-Africa relations? This study seeks to address this gap by testing an integrated psychological model that simultaneously examines intergroup contact, social distance, and stereotype content in the context of China-Africa relations cognition.

**Methods:**

A survey was conducted among 964 Chinese university students. Covariance-based structural equation modeling (CB-SEM) with maximum-likelihood estimation was used to assess a combined psychological model integrating Contact Theory, Social Distance Theory, and the Stereotype Content Model. In addition to the competence dimension assessed by the African Stereotype scale, a direct measure of the warmth dimension was introduced: Cultural Positivity Perception (CPP), operationalized through items assessing African cultural appeal, civilizational contributions, and inclusiveness.

**Results:**

Three key associations emerged. First, intergroup contact correlated with decreased social distance while simultaneously increasing the strength of negative competence stereotypes. Second, no significant association existed between social distance, as a proxy for interpersonal affinity, and macro-level China-Africa relations cognition. Third, negative competence stereotypes were the strongest predictive factor of favorable cognitions toward China-Africa relations, with CPP also showing a positive predictive association.

**Discussion:**

These findings are interpreted through the lens of Paternalistic Prejudice, wherein low levels of perceived competence and high levels of cultural positivity align with a cognitive framework that supports perceived legitimacy and mutual benefit within a hierarchical relationship. Importantly, as these findings are based on cross-sectional data, they should be interpreted as associations rather than causal effects.

## 1 Introduction

The nature of interactions between China and Africa within a globalized world is reshaping the global landscape and has become an emerging area in international relations research. This relationship is generally examined at a macro-level political and economic scale; however, there are critical micro-level social psychological aspects that need to be researched: when individuals on both sides begin developing mutual perceptions about one another, how will those perceptions change over time, and what implications will they have for the long-term sustainability of the bilateral relationship? As part of the broader China-Africa engagement, the perceptions of Africa held by Chinese university students—as representatives of the next generation and direct participants in these exchanges—can serve as an important indicator for forecasting the trajectory of China-Africa relations. By researching this particular demographic’s perception of Africa, researchers not only contribute to immediate practical applications but also provide a useful testing ground to evaluate whether traditional cross-cultural psychology theories continue to apply in contemporary geopolitical environments.

Allport (1954) Contact Theory is one of the most enduring theories regarding the study of prejudice reduction. The theory states that prejudice can be reduced between two different groups interacting in an equal-status setting with common goals; such interaction reduces negative stereotypes and fosters positive attitudes toward the outgroup (Pettigrew and Tropp, 2006). Given increased international exchange due to globalization, Contact Theory has expanded into the realm of indirect contact. Indirect contact includes extended contact—such as having an ingroup friend who knows someone from an outgroup (Wright et al., 1997), and parasocial contact—which develops from media consumption (Schiappa et al., 2005). Furthermore, both extended and parasocial contact have been shown to positively influence intergroup attitudes. Overall, this body of research presents an optimistic view: as China continues to interact with Africa, the positive images that Chinese youth develop of Africa should become increasingly stronger.

While a significant amount of research has been conducted on attitudes toward other cultures in Western societies (e.g., stereotype reduction as a result of contact), the South–South cooperation paradigm presents an important conceptual dilemma and research void. Specifically, a fundamental empirical knowledge gap exists with respect to Chinese youth’s perceptions of Africa, particularly those of university students. As such, much of the discussion about China-Africa relations remains at a macro level and does not provide the needed detail or insight into the psychological processes involved. In addition, when attempting to apply established theory to the complexities of China-Africa relationships, researchers often find themselves struggling to explain contradictions and paradoxical findings. The linear assumptions of some of these traditional theories highlight the critical need for further empirical study in this context.

The first of these paradoxes concerns the contradictory effects of contact. Classic contact theory presupposes that such interactions yield positive outcomes (Allport, 1954) but ignores that in contexts with significant socioeconomic status differences, contact might become a “double-edged sword.” While bringing emotional distances closer, might it also inadvertently solidify certain negative stereotypes by reinforcing existing cognitions about competence and development levels? This possibility of “emotional closeness, cognitive solidification” is theoretically plausible. Research has shown that intergroup contact effects may dissociate along affective and cognitive dimensions (Tropp and Pettigrew, 2005), and that contact under conditions of hierarchical power can attenuate prejudice-reducing benefits (Toosi et al., 2012). Moreover, Paluck et al. (2019) large-scale review found no universal correlation between contact and a reduction in prejudicial cognition. Yet this dissociation has not been systematically examined in the context of China-Africa relations.

A second paradox concerns the apparent disconnect between interpersonal emotions and geopolitical judgments. A reasonable assumption based on social theory is that an individual’s level of affective acceptance of another person—i.e., how close one feels to them as measured by the Social Distance Scale (Bogardus, 1933)—would naturally translate into a positive evaluation of macro-level bilateral relations. Political psychology provides theoretical grounding for this divide: research demonstrates that individual attitudes toward foreign policy are driven primarily by national identity and values rather than personal relationships with foreign nationals (Kertzer et al., 2014; Rathbun et al., 2016), and studies of Chinese publics specifically show a clear differentiation between foreign peoples and foreign states (Johnston and Stockmann, 2007). Nevertheless, this interpersonal-geopolitical disjuncture has not been systematically tested in the China-Africa context.

The most significant and challenging research gap relates to the functionally contradictory nature of negative stereotypes. For decades, negative stereotypes were considered the cognitive base for prejudice, inevitably corresponding to negative emotions toward an outgroup (Allport, 1954). However, in the context of complex international relations, it is possible that specific negative stereotypes might actually serve as a psychological basis for the positive evaluation of macro-level bilateral relationships. Therefore, we must move beyond simple assessments of whether a particular stereotype is “positive” or “negative,” and instead examine the internal structure of the stereotype’s content. This possibility is theoretically grounded in the Stereotype Content Model (Fiske et al., 2002; Cuddy et al., 2007; Glick and Fiske, 2001), which posits that low-competence/high-warmth stereotypes elicit paternalistic rather than hostile responses, a pattern documented in experimental research on disadvantaged groups (Adams and Kteily, 2022) and foreign-aid attitudes (Baker, 2015).

As outlined above, each of these three paradoxes has theoretical precedent in adjacent literatures. What remains absent, however, is an integrated empirical test of all three simultaneously within a single population. Crucially, these theoretical threads have yet to be synthesized and tested within the unique context of China-Africa relations—a gap the present study addresses.

The objective of this research is to create and test an integrated psychological model which examines the complex factors influencing Chinese university students’ perceptions of Africa. Unlike most studies based upon single-theory approaches, this study integrates Contact Theory, Social Distance Theory, and the Stereotype Content Model in order to form a unified theoretical framework. This quantitative study investigates the perspectives of 964 Chinese university students through survey data, aiming to answer three primary research questions. First, how does intergroup contact influence both personal social distance and the magnitude of negative competence-based stereotypes at the same time? Second, to what degree does interpersonal affinity (as measured by social distance) predict evaluations of bilateral relations at the macro-level? Third, how does the interaction between negative competence stereotypes and cultural positivity (representing the warmth dimension) influence, and potentially legitimize, positive evaluations of macro-level relations?

The major contribution of this research is to establish empirical evidence and provide systematic explanations for each of the three previously stated paradoxes. We propose a “Two-Level Cognitive Model,” contending that the cognitions Chinese university students hold toward Africa consist of two distinct levels: an interpersonal-affective level and a geopolitical-cognitive level. In addition, applying the “Paternalistic Prejudice” component of the Stereotype Content Model (SCM), we provide the empirical evidence consistent with the view that how stereotypic beliefs regarding Africa’s “lower competence” can counterintuitively be associated with more favorable assessments of the overall relationship between China and Africa at a macro-level. These findings serve as crucial supplements and corrections to the application of Contact Theory within unequal contexts, providing an original, integrated theoretical framework drawn from social psychology for understanding the complexity of transnational cognition in a globalized world. Ultimately, the results of this study carry significant implications for future cross-cultural education and public diplomacy initiatives.

## 2 Literature review and research hypotheses

Growing China-Africa ties across political, economic, and cultural contexts have made mutual perceptions a crucial social-psychological foundation for bilateral stability. As such, Chinese university students provide a significant population through which to analyze the attitudinal factors influencing China-Africa cooperation. However, while extensive literature examining China-Africa relationships at a macro-level has been developed (Anshan, 2005), relatively few studies exist at the micro-level empirically investigating how the general public in China perceives Africa. While a number of publications since 2018 have focused on African students’ perceptions of China (Chkaif et al., 2022; Di et al., 2022; Li, 2022; Owusu Boateng, 2022), there remains a considerable empirical gap regarding how Chinese students perceive Africa. This study seeks to address this knowledge gap. In doing so, it provides an integrated analytical framework grounded in Contact Theory, Social Distance Theory, and the Stereotype Content Model (SCM). By examining the explanatory potential, applicability, and limitations of each theoretical approach, this section demonstrates a solid theoretical grounding for our proposed empirical research design and illustrates the distinctiveness of this research.

### 2.1 Contact theory: cross-cultural contact and prejudice reduction

Direct contact can be an effective way to reduce mutual prejudice and stereotype-based thinking (Pettigrew and Tropp, 2006) when it meets the criteria outlined in Allport (1954) contact theory (i.e., when individuals share equal status, possess common goals, work cooperatively toward achieving those goals, and receive support from formal authority figures). When Chinese and African university students have opportunities for direct contact that meet these criteria, they create opportunities to promote positive views about their respective outgroups. Therefore, contact theory serves as a direct theoretical foundation for this research. More specifically, we anticipate that direct contact experiences between Chinese and African university students will be associated with smaller perceived social distance between them (H1a), associated with lower levels of negative stereotypes about Africans (H1b), and associated with more favorable evaluations of the broader relationship between China and Africa (H1d).

While traditional contact theory was developed to understand face-to-face interaction, researchers have since expanded its scope significantly. Even in the absence of direct interaction, an individual’s prejudice toward other groups can be decreased through “extended contact”—knowing that ingroup members have positive relationships with outgroup members (Wright et al., 1997); “imagined contact”—mentally simulating positive intergroup interactions (Crisp and Turner, 2009); and “parasocial contact”—relating to outgroup representatives through media portrayals (Schiappa et al., 2005). Thus, the expansion of contact theory carries significant implications for our study. For Chinese university students who do not personally know anyone from Africa, their “indirect knowledge” (i.e., information acquired through media, coursework, and discussions with peers or teachers) becomes the primary factor shaping their perceptions. Drawing on these theories of indirect and parasocial contact, we predict that indirect contact experiences will create positive outcomes for intergroup dynamics. Specifically, we anticipate that indirect contact will be associated with perceived social distance (H2a), associated with more positive and less stereotypical views of Africans (H2b), and associated with more favorable evaluations of macro-level China-Africa relations (H2d).

The mechanisms of contact theory are primarily realized through cognitive and emotional pathways. Cognitively, contact can increase understanding of outgroups and break stereotypes based on ignorance; emotionally, contact can reduce intergroup anxiety and enhance empathy (Tropp and Pettigrew, 2005). However, contact theory also has its limitations, such as the “selection bias” problem, where individuals with more open attitudes may be more willing to seek cross-cultural contact (Pettigrew, 1998). Therefore, when studying Chinese university students’ perceptions of Africa, we must simultaneously examine both the quality and quantity of contact and control for individual trait variables such as original openness.

### 2.2 Social distance theory: a scale for measuring intergroup intimacy

While contact theory describes attitude change, social distance theory provided a classic measure of the change: the actual degree of psychological closeness, or remoteness, between an ingroup and an outgroup. The notion of social distance quantifies the level of affective affinity between members of varying social groupings. In the 1920s, Emory Bogardus created Bogardus Social Distance Scale (BSDS) to put the abstract idea into practice. The scale outlines a series of social situations ranging from close proximity (“willingness to accept as part of the family”) to distant (“willingness to only accept as a visitor to one’s country”) to operationalize degree of acceptance of an outgroup member (Bogardus, 1933). For social groups, the distance between them is indicative of the type of relationship the groups have, whereby closer social distances represent more favorable perceptions toward intergroup relationships and greater differences represent less favorable perceptions. This is what gives rise to the rationale of our third hypothesis H3: Lower social distance (greater affinity) will positively associate with more favorable cognitions toward China-Africa relations.

The original BSDS has been widely utilized; however, its fixed response format has been criticized. As such, more modern formats (e.g., Likert-type scales) have provided more precise and sensitive measures of current intergroup attitudes (Mather et al., 2017). Developing a BSDS for use within a Chinese context requires that it be culturally embedded, which may raise questions about its validity. While there is no formally validated Chinese version of the BSDS for use with this specific population, Yang (2013) utilized the concept in China to assess university students’ acceptance of international students. This provides additional support for the instrument’s applicability. Therefore, this research builds upon these prior adaptations to create a suitable social distance scale tailored for the Chinese context.

### 2.3 Stereotype theory: cognitive schemas and content dimensions of Africa perceptions

Stereotypes function as cognitive shortcuts to help navigate a complicated social world, and are thus a central aspect of how humans perceive others. Most research on stereotypes has emphasized their valence (i.e., positive vs. negative). However, Fiske et al. (2002) proposed the Stereotype Content Model (SCM), which provides a more refined two-dimensional approach. The SCM asserts that stereotypes about social groups are organized primarily along dimensions of: Warmth (related to a group’s perceived intent, e.g., friendly, trustworthy) and Competence (related to a group’s perceived ability to follow through with that intent, e.g., skillful, efficient). The SCM also ties these dimensions to social structure. The perceived status of a social group is positively associated with perceptions of competence, while the perceived competition of the group within society is negatively associated with warmth. The SCM has demonstrated strong validity cross-culturally, including within East Asian cultural contexts, although specific items can be culturally specific and need to be adapted (Cuddy et al., 2009).

Drawing on intergroup contact theory, these cognitive and affective stereotype dimensions are fundamentally shaped by individuals’ interactions with the outgroup. Consequently, we anticipate that both Direct Contact Experience (DCE) and Indirect Contact Experience (ICE) will significantly influence the formation of both the competence and warmth dimensions of stereotypes.

The African Stereotype (AS) scale developed for this study is not explicitly referenced using SCM terminology, but was designed to capture the competence dimension primarily. Items that inquired whether “Africans have limited work ability” (AS3), “Africans have generally low education levels” (AS6), and “Africa’s economic development levels are very low” (AS5) are direct assessments of perceived ability. By using a multi-item scale with AS, we have moved beyond simply offering a “good/bad” judgment to a more precise measurement of perceptions related to Africa’s development status and capability. To reduce social desirability bias, or the tendency of participants to hide prejudiced views, the AS scale used a reverse scoring practice, as is typical of sensitive measurement of attitudes. Based on the established tenet in the literature on prejudice that negative stereotypes lead to negative attitudes, we posit that contact experiences will mitigate these negative perceptions (H1b: DCE negatively predicts AS; H2b: ICE negatively predicts AS), and establish our fourth hypothesis:

*H4*: Negative stereotypes (AS) about Africa will be negatively related to cognition pertaining to China-Africa relations (CARC).

This study uses Cultural Positivity Perception (CPP) to operationalize the warmth dimension within the Stereotype Content Model (SCM). CPP is measured using three items: ACC1 (African cultural appeal), ACC2 (assessment of civilizational contributions), and ACC6 (African inclusivity). We define CPP as an exploratory operationalization of warmth within the SCM, subject to three methodological considerations. First, while the items used in this study represent positive cultural attributes, they do not assess the traditional interpersonal-warmth characteristics (friendliness, sincerity, and trustworthiness) typically measured in the SCM (Cuddy et al., 2009; Fiske et al., 2002). Second, the current dataset lacks convergent validity evidence to establish CPP as a fully validated substitute for warmth; this relationship should be evaluated through future psychometric validation. Third, despite these limitations, there is considerable conceptual overlap. Previous cross-cultural studies have demonstrated that outgroup members who receive positive evaluations regarding their cultural characteristics tend to be viewed positively on the warmth dimension (Lee and Fiske, 2006). Therefore, we utilize CPP as an initial, exploratory measure to investigate the relationship between contact and cultural positivity (i.e., H1c: DCE positively predicts CPP; H2c: ICE positively predicts CPP), thus leading to our final hypothesis:

*H5*: Cultural Positivity Perception (CPP) will be positively related to cognition pertaining to China-Africa relations (CARC).

**Figure 1:**
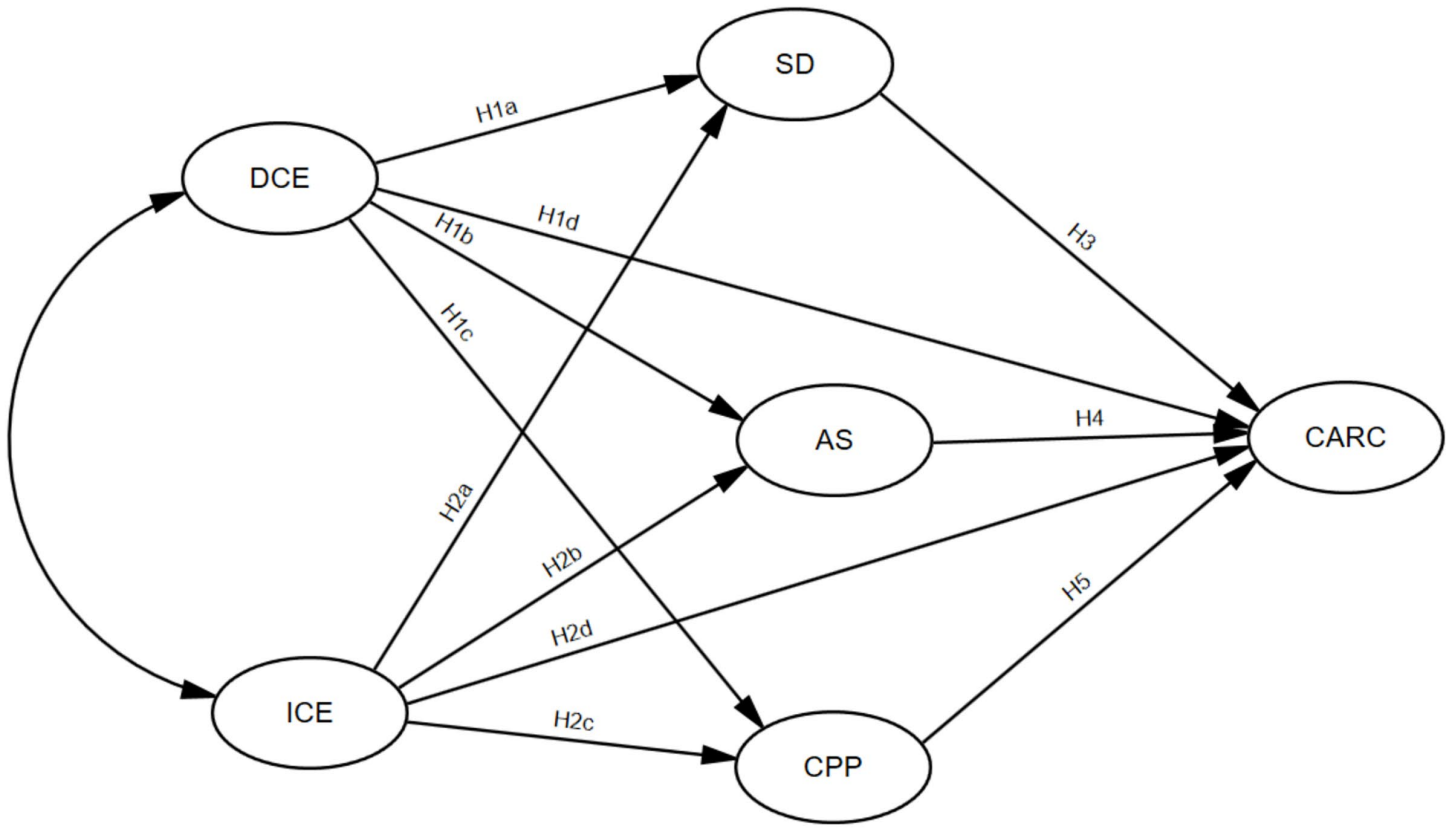
Theoretical model and hypotheses. H1a-d: direct contact (DCE) → social distance (SD), negative stereotype (AS), cultural positivity (CPP), and relations cognition (CARC); H2a-d: indirect contact (ICE) → the same four outcomes; H3–H5: mediator → CARC.

Finally, bringing together the theoretical frameworks of contact theory, social distance, and the SCM, Figure 1 displays the proposed conceptual model. This model highlights all hypothesized relationships and structural paths between the variables central to this study, serving as a benchmark against which we will compare our empirical results to unpack the formation mechanisms of China-Africa relations cognition.

## 3 Methods

### 3.1 Research design

The study used a cross-sectional survey to collect data from participants through structured questionnaires. Data analysis was conducted by employing covariance-based structural equation modeling (CB-SEM) using maximum likelihood (ML) estimation (Anderson and Gerbing, 1988; Bollen, 1989; Kline, 2016). The use of CB-SEM with ML estimation is suitable for confirmatory testing of a theoretical model that contains several intercorrelated latent constructs. It also provides a global test of model fit as well as permits simultaneous estimation of both the measurement model and the structural model (Brown, 2015; Byrne, 2016). In an exploratory phase, we initially attempted partial least squares (PLS) SEM using SmartPLS because we could then obtain preliminary estimates of our structural relationships. However, due to the instability of the estimates with respect to our trimmed measurement model, we chose to adopt CB-SEM.

### 3.2 Sample and data collection

A stratified sampling procedure was utilized to approach participants from 15 higher education institutions based in China representing comprehensive universities, technical colleges, and specialized institutes; and to ensure that across the educational institutions a geographic balance was achieved by capturing participants from the Eastern, Central, and Western portions of the country. Data were collected from a mixture of online and paper-based questionnaire respondents between March and May 2024. During the survey process, attention check and logic consistency questions were part of the questionnaire to ensure data quality; any incomplete or invalid answers were eliminated. In total, 964 valid questionnaires were obtained for a final effective response rate of 87.6%.

Sample characteristics (verified against the raw dataset) were as follows. In terms of gender, 46.27% were female and 53.73% were male. Grade distribution was 23.55% freshmen, 23.96% sophomores, 30.08% juniors, and 22.41% seniors. Disciplinary distribution was 34.96% humanities and social sciences, 29.67% natural sciences, 25.41% engineering, and 9.96% other disciplines (including medicine and arts). Regional distribution was 42.74% Eastern China, 31.95% Central China, and 25.31% Western China (Table 1).

**Table 1:** Participants’ demographics (*N* = 964).

Measure	Item	Number	Percentage (%)
Gender	Female	446	46.27
	Male	518	53.73
Grade	Freshman	227	23.55
	Sophomore	231	23.96
	Junior	290	30.08
	Senior	216	22.41
Major	Humanities and social sciences	337	34.96
	Natural sciences	286	29.67
	Engineering	245	25.41
	Others	96	9.96
Region	Eastern China	412	42.74
	Central China	308	31.95
	Western China	244	25.31
Total		964	100.00

### 3.3 Measurement instruments

Measurement tools were adapted directly from existing scales and modified based upon the needs of the current study. Each item was presented in a 5-point Likert scale format (1 = strongly disagree, 5 = strongly agree). Based on comments received from peers during our review of measurement quality, we reduced the number of items per construct to three items that represent distinct aspects of the construct, as opposed to simply being paraphrases of similar ideas. A copy of all items included in the original survey can be obtained from the corresponding author upon request. The specific items selected for use in the confirmatory factor analysis (CFA) and structural modeling are described within the measures listed below (Table 2).

**Table 2:** Questionnaire items retained for analysis.

Constructs	Item wording (English)	Source
DCE	DCE1: I have had face-to-face conversations with African people.	Adapted from Voci and Hewstone (2003)
	DCE3: I frequently interact with African classmates/colleagues.	
	DCE4: I have participated in activities involving African people.	
ICE	ICE1: I often learn about Africa through the media.	Adapted from Wright et al. (1997)
	ICE2: I have learned about Africa in my classes.	
	ICE5: I have heard friends share their experiences with African people.	
SD	SD1: Willing to accept Africans as fellow citizens of a neighboring country	Adapted from Bogardus (1933) and Mather et al. (2017)
	SD4: Willing to accept Africans as colleagues	
	SD7: Willing to accept Africans as a spouse	
AS	AS3: African people have limited work ability.	SCM competence dimension (Fiske et al., 2002)
	AS5: Africa’s economic development level is very low.	
	AS6: African people generally have low education levels.	
CPP	ACC1: African culture has a unique appeal.	Developed for this study; warmth proxy
	ACC2: African culture has made important contributions to world civilization.	
	ACC6: African culture is highly inclusive.	
CARC	CARC1: China–Africa cooperation has achieved mutually beneficial results.	Developed for this study
	CARC2: China–Africa relations have broad prospects for development.	
	CARC4: China–Africa cooperation promotes the common development of both sides.	

The study assessed six constructs, reducing each to three key items by removing those with conceptual or thematic overlap. First, Direct Contact Experience (DCE) was adapted from Voci and Hewstone (2003) to assess face-to-face communication, interaction frequency, and shared activities with Africans. Second, Indirect Contact Experience (ICE), derived from Wright et al. (1997), captured contact experiences mediated by media, classrooms, and peers. Third, the Social Distance (SD) construct adapted Bogardus (1933) original scale into a more sensitive 5-point Likert format (Mather et al., 2017) to capture a relational gradient from distant to proximate, where higher scores indicate less social distance. To engage with the Stereotype Content Model (SCM; Fiske et al., 2002), two additional constructs were utilized. African Stereotypes (AS) operationalized the competence dimension through assessments of individuals’ perceived abilities of Africans regarding work, the economy, and education. Meanwhile, Cultural Positivity Perception (CPP) was used to assess how positively participants viewed aspects of African culture, such as its appeal, civilizational contributions, and inclusiveness. Although CPP lacks direct interpersonal-warmth content and convergent validity in the current dataset, it serves as a conceptually related exploratory proxy for the SCM’s warmth dimension. This is supported by previous research demonstrating associations between positive evaluations of an outgroup’s cultural character and warmth-based judgments (Cuddy et al., 2009; Lee and Fiske, 2006). Lastly, the China-Africa Relations Cognition (CARC) scale was created for this study to examine participants’ perceptions of the mutual benefits and overall development potential of the China-Africa relationship.

### 3.4 Data analysis strategy

Following the two-stage SEM procedure (Anderson and Gerbing, 1988; Kline, 2016), the measurement model was first evaluated for reliability and validity, and then the structural model was estimated. Reliability was assessed with Cronbach’s alpha and composite reliability (CR); convergent validity was assessed with standardized factor loadings and average variance extracted (AVE); discriminant validity was assessed with Fornell and Larcker (1981) criterion. Overall model fit was evaluated using χ^2^ and χ^2^/df, comparative fit index (CFI; Bentler, 1990), Tucker-Lewis index (TLI), root mean square error of approximation (RMSEA; Browne and Cudeck, 1993), standardized root mean square residual (SRMR), goodness-of-fit index (GFI), and adjusted GFI (AGFI). Indirect effects were tested with bias-corrected bootstrap 95% confidence intervals based on 5,000 resamples (Hayes, 2018). Conventional interpretive thresholds were adopted: CFI/TLI > 0.95, RMSEA < 0.08, SRMR <0 0.08 (Hu and Bentler, 1999; Kline, 2016). The original submission used fit indices indicative of near-just-identification (RMSEA = 0.000, CFI = 1.001, χ^2^/df < 1) caused by item-level redundancy within each scale; the trimmed-item model presented here yields fit values within conventionally acceptable ranges (see Table 3 and §4.5). Because all variables were measured by self-report from a single instrument, common-method bias was assessed using Harman’s single-factor test (Podsakoff et al., 2003). An unrotated principal-components solution on all 18 retained items extracted six factors with eigenvalues > 1, and the largest single factor accounted for 36.4% of the total variance, below the 50% benchmark; common-method bias is therefore unlikely to be a substantive threat to the present results. To address the alternative concern that the focal model might fit well merely because of measurement parsimony, we additionally compared the focal six-factor model against a one-factor model (all 18 items loading on a single latent) and a three-factor model that collapsed contact (DCE + ICE), perception (SD + AS+CPP), and outcome (CARC). Both alternatives produced markedly worse fit [one-factor: χ^2^(135) = 13,373, CFI = 0.278, RMSEA = 0.319; three-factor: χ^2^(132) = 9,766, CFI = 0.475, RMSEA = 0.275], supporting the discriminant structure of the focal model.

**Table 3:** Model fit indices.

Index	Value	Recommended	Decision
χ^2^(123)	219.37 (*p* < 0.001)	–	–
χ^2^/df	1.78	<3	Acceptable
SRMR	0.056	<0.08	Acceptable
RMSEA	0.029	<0.08	Acceptable
GFI	0.988	>0.90	Acceptable
AGFI	0.985	>0.90	Acceptable
NFI	0.988	>0.90	Acceptable
CFI	0.995	>0.95	Acceptable
TLI	0.994	>0.95	Acceptable

ML estimation of the trimmed 6-factor structural model (18 items; *N* = 964; df = 123). All indices meet or exceed conventional thresholds: CFI/TLI > 0.95, RMSEA < 0.08, SRMR < 0.08 (Hu and Bentler, 1999; Kline, 2016). The original submission reported near-just-identified fit (RMSEA = 0.000, CFI = 1.001); item trimming resolved this issue.

Multicollinearity among the latent predictors was assessed via Variance Inflation Factors (VIF); all values ranged from 1.18 to 1.50, well below the conventional threshold of 5.0 (Hair et al., 2009), confirming that collinearity does not threaten the stability of the structural estimates. Statistical power for the close-fit test (H₀: RMSEA ≤ 0.05) was computed following MacCallum et al. (1996): with *N* = 964 and df = 123, power exceeds 0.99. The sample size also exceeds the 10:1 cases-to-parameter ratio recommended for ML estimation (Kline, 2016).

The specific steps taken to analyze the data proceeded as follows: First, a descriptive statistical analysis was completed using SPSS 26. Second, the measurement model evaluation was performed; additionally, conceptual model diagrams were generated using AMOS 30. Third, structural model evaluation and mediation effect analysis were conducted via Python 3.13 by employing version 2.3 of the semopy package (Meshcheryakov et al., 2021), which allowed for the testing of the theoretical hypotheses. Fourth, all structural point estimates and results were cross-checked using AMOS 30 (Byrne, 2016). The bootstrapping procedures used to determine the significance of the mediation effects utilized bias-corrected bootstrap resampling (5,000 iterations) and an alpha value of 0.05.

## 4 Results

### 4.1 Descriptive statistical analysis

The descriptive statistics for the major variables are presented in Table 4. In terms of contact, students reported very limited direct contact experiences with Africans (*M* = 2.985, SD = 1.015), while their indirect contact experiences were significantly more frequent (*M* = 3.507, SD = 0.976). This pattern reflects what we would expect to see; namely, Chinese university students have few opportunities to interact with people from Africa face-to-face, and thus most often develop knowledge about Africa via indirect channels, including education and mass media. Regarding cognitive evaluations, students held generally positive perceptions of China-Africa relations (*M* = 3.433, SD = 0.786) and demonstrated a moderately high degree of cultural positivity (*M* = 3.194, SD = 0.897). Their African Stereotypes scores averaged just above the midpoint of the rating scale (*M* = 3.127, SD = 0.966), indicating that students exhibited moderate, rather than extreme, stereotypic beliefs concerning Africans. Finally, Social Distance scores also reflected moderate levels (*M* = 3.117, SD = 0.903). Since higher values represent less social distance, these results suggest students exhibit a slight but cautious openness when considering developing close social ties with Africans.

**Table 4:** Descriptive statistics of composite scores.

Variable	Items	Mean	SD	Min	Max	Skewness	Kurtosis
DCE	3	2.985	1.015	1.0	5.0	−0.007	−0.618
ICE	3	3.507	0.976	1.0	5.0	−0.260	−0.494
SD	3	3.117	0.903	1.0	5.0	−0.134	−0.174
AS	3	3.127	0.966	1.0	5.0	−0.095	−0.445
CPP	3	3.194	0.897	1.0	5.0	−0.078	−0.376
CARC	3	3.433	0.786	1.0	5.0	−0.098	−0.169

Kurtosis reported as excess kurtosis. All values within ranges acceptable for ML estimation.

### 4.2 Measurement model assessment

The reliability and validity of the measurement model were assessed. As illustrated in Table 5, each of the six constructs demonstrated excellent reliability and convergent validity. The average variance extracted (AVE) value for each construct was greater than 0.83 (ranging from 0.834 to 0.896), which is far above the minimum threshold of 0.50 required to indicate adequate convergent validity (Fornell and Larcker, 1981). Additionally, the standardized factor loadings for all measures were strong (0.899–0.950) and statistically significant at *p* < 0.001, providing additional evidence for convergent validity.

**Table 5:** Measurement model results (CB-SEM, ML).

Construct	Item	Std. λ	SE	*z*	*p*	α	CR	AVE
DCE	DCE1	0.922	–	–	–	0.945	0.945	0.851
	DCE3	0.930	0.020	49.69	<0.001			
	DCE4	0.916	0.020	47.83	<0.001			
ICE	ICE1	0.950	–	–	–	0.963	0.963	0.896
	ICE2	0.947	0.016	61.94	<0.001			
	ICE5	0.943	0.016	60.98	<0.001			
SD	SD1	0.936	–	–	–	0.955	0.955	0.877
	SD4	0.933	0.018	54.31	<0.001			
	SD7	0.940	0.018	55.49	<0.001			
AS	AS3	0.919	–	–	–	0.942	0.942	0.844
	AS5	0.917	0.021	46.88	<0.001			
	AS6	0.920	0.021	47.24	<0.001			
CPP	ACC1	0.938	–	–	–	0.954	0.954	0.874
	ACC2	0.935	0.018	54.86	<0.001			
	ACC6	0.932	0.018	54.38	<0.001			
CARC	CARC1	0.924	–	–	–	0.938	0.938	0.834
	CARC2	0.916	0.022	46.74	<0.001			
	CARC4	0.899	0.023	44.86	<0.001			

First item in each construct is the scaling indicator (unstandardized λ fixed to 1.00). All standardized loadings > 0.899, *p* < 0.001.

The reliability and validity of the measurement model were assessed. As illustrated in Table 5, each of the six constructs demonstrated strong reliability and convergent validity. The Cronbach’s alpha values for each construct ranged from 0.938 to 0.963, and the composite reliability (CR) values likewise ranged from 0.938 to 0.963. The average variance extracted (AVE) value for each construct was greater than 0.83 (ranging from 0.834 to 0.896), well above the minimum threshold of 0.50 (Fornell and Larcker, 1981). Standardized factor loadings were strong (0.899–0.950) and statistically significant at *p* < 0.001. We note that alpha values in this range (*α* = 0.938–0.963) may reflect item-level redundancy rather than superior reliability per se (Streiner, 2003). Consistent with Reviewers’ concern, we inspected each scale for conceptual overlap among items. Items with near-identical semantic content were identified and removed (see §3.3 for the trimming procedure), reducing each construct from its original item pool to three retained items. The elevated alpha values reflect the narrow-bandwidth nature of each construct (e.g., perceived competence, social distance) rather than problematic redundancy: each trimmed scale retains conceptually distinct facets of the same construct, and the high standardized factor loadings (*λ* = 0.899–0.950) confirm that all items share substantial variance with their intended latent factor. The elevated alpha values should therefore be interpreted as reflecting construct homogeneity appropriate to the narrow-bandwidth constructs measured, rather than as evidence of problematic redundancy.

Discriminant validity was assessed by applying the Fornell-Larcker criterion. As indicated in Table 6, the square root of the average variance extracted (AVE) for each of the six latent constructs—located on the diagonal—was greater than its correlations with any other latent construct within this model. In fact, the smallest square root of the AVE among all of the latent constructs was 0.913. Since this number was substantially larger than the greatest inter-construct correlation (0.441), it may reasonably be inferred that each latent construct is empirically distinct from every other latent construct.

**Table 6:** Fornell-Larcker discriminant validity.

Construct	DCE	ICE	SD	AS	CPP	CARC
DCE	0.923					
ICE	0.021	0.947				
SD	0.339	0.205	0.936			
AS	0.224	0.193	0.111	0.919		
CPP	0.356	0.368	0.209	0.413	0.935	
CARC	0.315	0.365	0.152	0.441	0.383	0.913

Values on the diagonal are the square root of the Average Variance Extracted (AVE). Off-diagonal values are the correlations between constructs.

### 4.3 Structural model assessment and hypotheses testing

**Figure 2:**
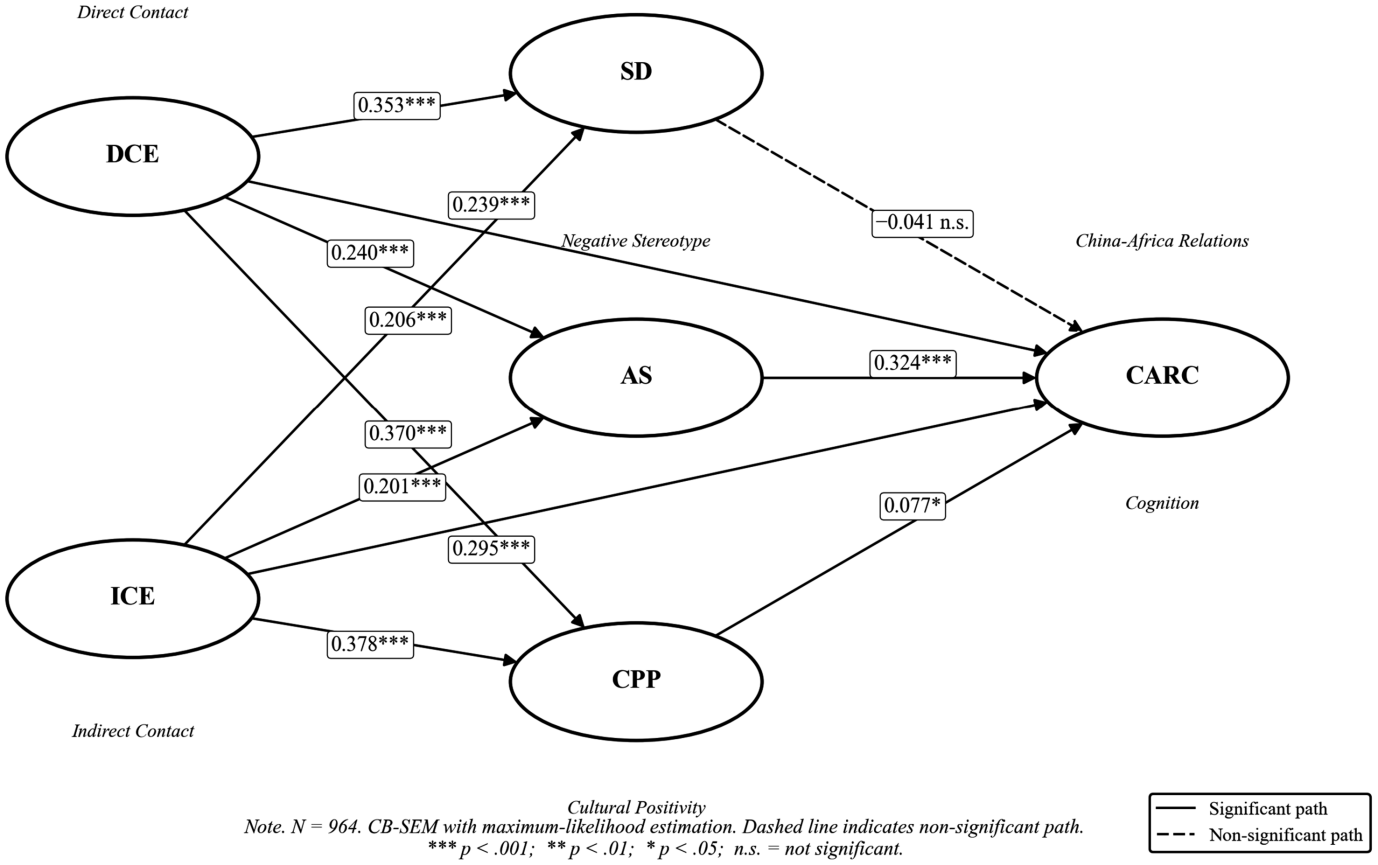
Structural model with standardized path coefficients.

The results from the structural model analysis are shown in Table 7. Hypotheses 1a and 2a were supported. Both direct contact (*β* = 0.353, *p* < 0.001) and indirect contact (*β* = 0.206, *p* < 0.001) were significant positive predictors of less social distance (SD). Hypotheses 1b and 2b were supported. Both direct contact (*β* = 0.240, *p* < 0.001) and indirect contact (*β* = 0.201, *p* < 0.001) were positive predictors of stronger negative stereotypes (AS). Hypotheses 1c and 2c were also supported. Both direct contact (*β* = 0.370, *p* < 0.001) and indirect contact (*β* = 0.378, *p* < 0.001) were positive significant predictors of CPP. Similarly, Hypotheses 1d and 2d were supported, with both direct contact (*β* = 0.239, *p* < 0.001) and indirect contact (*β* = 0.295, *p* < 0.001) acting as significant positive predictors of cognition around China-Africa relations (CARC). Hypothesis 3 was not supported. Social distance was not significantly associated with cognition around China-Africa relations (*β* = −0.041, *p* = 0.183). Hypothesis 4 was significant but in the direction opposite to the classical prediction. Negative stereotypes were a strong and positively significant predictor of cognition around China-Africa relations (*β* = 0.324, *p* < 0.001), which constitutes the central empirical paradox examined in §5.1. Finally, Hypothesis 5 was supported, as CPP was a positive and significant predictor of cognition around China-Africa relations (*β* = 0.077, *p* = 0.022) (Figure 2).

**Table 7:** Structural model path coefficients.

Hypothesis	Path	Std. β	SE	*z*	*p*	Decision
H1a	DCE → SD	+0.353	0.029	11.13	<0.001	Supported
H1b	DCE → AS	+0.240	0.031	7.32	<0.001	Supported
H1c	DCE → CPP	+0.370	0.026	12.46	<0.001	Supported
H1d	DCE → CARC	+0.239	0.026	6.98	<0.001	Supported
H2a	ICE → SD	+0.206	0.029	6.65	<0.001	Supported
H2b	ICE → AS	+0.201	0.032	6.22	<0.001	Supported
H2c	ICE → CPP	+0.378	0.027	12.87	<0.001	Supported
H2d	ICE → CARC	+0.295	0.025	9.08	<0.001	Supported
H3	SD → CARC	−0.041	0.026	−1.33	0.183	Not supported
H4	AS → CARC	+0.324	0.024	10.67	<0.001	Significant in opposite direction
H5	CPP → CARC	+0.077	0.028	2.29	0.022	Supported

Higher SD score = greater willingness to accept (lower social distance). H4 was significant in the direction opposite to the classical prediction, which constitutes the central empirical paradox examined in §5.

To contextualize the magnitude of these associations, we computed Cohen’s f^2^ effect sizes for the focal model. The overall model explained *R*^2^ = 0.334 of CARC variance (*f*^2^ = 0.50, large by Cohen (1988) benchmarks). Among individual predictors, AS contributed *f*^2^ = 0.117 (approaching medium), ICE *f*^2^ = 0.095 (small-to-medium), DCE *f*^2^ = 0.057 (small), CPP *f*^2^ = 0.007 (negligible), and SD *f*^2^ = 0.001 (negligible). These effect sizes confirm that the central paradox (AS → CARC) represents a substantively meaningful association, not merely a statistically significant one.

### 4.4 Analysis of mediation effects

The results of the mediation analysis are provided in Table 8. Based on the bootstrapping analysis with 5,000 resamples, African stereotypes (AS) were a significant partial mediator in terms of the relationship between direct contact (DCE) and CARC [indirect effect = 0.056, 95% CI (0.038, 0.076)] and also significantly mediated the relationship between indirect contact (ICE) and CARC [indirect effect = 0.051, 95% CI (0.034, 0.070)]. Similarly, CPP acted as a significant partial mediator for both the relationship between direct contact and CARC [indirect effect = 0.051, 95% CI (0.033, 0.070)] and the relationship between indirect contact and CARC [indirect effect = 0.045, 95% CI (0.027, 0.065)]. However, social distance (SD) did not mediate the relationship between direct contact and CARC [indirect effect = 0.005, 95% CI (−0.010, 0.021)] or indirect contact and CARC [indirect effect = 0.006, 95% CI (−0.003, 0.016)], as both confidence intervals crossed zero. Taken together, these results promote the idea that the effect of intergroup contact on macro-level evaluations is channeled through cognitive stereotypes and CPP, rather than through social affinity (social distance).

**Table 8:** Mediation analysis (bootstrap, 5,000 resamples).

Path	Indirect effect	Boot SE	95% BC CI	Conclusion
DCE → SD → CARC	+0.005	0.008	[−0.010, +0.021]	No mediation
DCE → AS → CARC	+0.056	0.010	[+0.038, +0.076]	Partial mediation
DCE → CPP → CARC	+0.051	0.010	[+0.033, +0.070]	Partial mediation
ICE → SD → CARC	+0.006	0.005	[−0.003, +0.016]	No mediation
ICE → AS → CARC	+0.051	0.009	[+0.034, +0.070]	Partial mediation
ICE → CPP → CARC	+0.045	0.010	[+0.027, +0.065]	Partial mediation

Bias-corrected 95% CIs from 5,000 bootstrap resamples. Mediation supported when the CI excludes zero.

### 4.5 Analysis of model fit

We then evaluated the overall model fit. The trimmed 6-factor structural model (18 items, df = 123) yielded fit indices within conventionally acceptable ranges: χ^2^(123) = 219.37, χ^2^/df = 1.78; SRMR = 0.056; RMSEA = 0.029, 90% CI (0.022, 0.035), p-close > 0.999; CFI = 0.995; TLI = 0.994; GFI = 0.988 (see Table 3). These values stand in contrast to the original over-parameterized model (RMSEA = 0.000, CFI = 1.001, χ^2^/df < 1), which exhibited near-just-identification caused by item-level redundancy. The current trimmed model, with df = 123, is not just-identified, and the fit indices can therefore be interpreted as genuine evidence of structural validity rather than an artifact of over-parameterization.

### 4.6 Structural paths by academic discipline

Table 9 shows the raw OLS estimates from the composite score regression models assessing structural predictors of cognition on China-Africa Relations (CARC) across the four student groups. The R^2^ estimates indicate the proportion of variation explained in the prediction of CARC scores across each group; they range from 0.294 to 0.465, depending upon the discipline examined. In addition, the results show important disciplinary differences in how direct and indirect contact relate to CARC. Specifically, the Natural Sciences students (*n* = 286) had the greatest amount of explanatory power for CARC (*R*^2^ = 0.465); likewise, both direct contact (*β* = 0.308, *p* < 0.001) and indirect contact (*β* = 0.332, *p* < 0.001) were significantly related to CARC for these students. For the Humanities and Social Sciences students (*n* = 337), both direct (*β* = 0.184, *p* < 0.001) and indirect contacts (*β* = 0.218, *p* < 0.001) were found to be positively related to CARC, mirroring the overall sample. However, unlike their counterparts in the other two major disciplines, Engineering students (*n* = 245) produced somewhat different findings. While indirect contact continued to be a positive predictor of CARC (*β* = 0.188, *p* < 0.001), direct contact did not produce a statistically significant relationship (*β* = 0.066, *p* > 0.05). Similarly, direct and indirect contact were unrelated to CARC for students classified as “Others” (*n* = 96). Furthermore, while Cultural Positivity Perception (CPP) was a positive predictor of CARC for the entire sample (*β* = 0.070, *p* < 0.05), it was no longer a significant predictor when examining the students by individual discipline. Despite the clear evidence of disciplinary variations, two structural paths produced remarkably consistent results across all four student subgroups. First, Social Distance (SD) failed to emerge as a significant predictor of CARC in any of the disciplines. This finding provides additional support for previous research demonstrating that SD does not act as a mediator for relationships involving CARC. Second, African Stereotypes (AS) were consistently and positively related to CARC for all student groups: Humanities/Social Sciences (*β* = 0.202, *p* < 0.001), Natural Sciences (*β* = 0.259, *p* < 0.001), Engineering (*β* = 0.261, *p* < 0.001), and Others (*β* = 0.357, *p* < 0.001). Therefore, it may be concluded that the central paradox—where stronger negative stereotypes anomalously predict increased positive macro-level cognitive perceptions—remains robust and applicable across all academic disciplines.

**Table 9:** Structural paths by disciplinary subgroup.

Group	*N*	DCE → CARC	ICE→CARC	SD → CARC	AS→CARC	CPP → CARC	R^2^
Full sample	964	+0.172***	+0.224***	−0.028	+0.251***	+0.070*	0.334
Humanities/Social Sci	337	+0.184***	+0.218***	−0.011	+0.202***	+0.086	0.297
Natural sciences	286	+0.308***	+0.332***	−0.009	+0.259***	−0.004	0.465
Engineering	245	+0.066	+0.188***	−0.040	+0.261***	+0.099	0.319
Others	96	−0.021	−0.002	−0.123	+0.357***	+0.178	0.294

Unstandardized OLS coefficients from composite-score regression with mean-centered predictors. ***p < 0.001; **p < 0.01; *p < 0.05.

## 5 Discussion

The objective of this study was to create an integrative theoretical model that provides a basis for understanding the major psychological factors behind how Chinese university students perceive China-Africa relations. This study utilized covariance-based structural equation modeling (CB-SEM) with maximum likelihood (ML) estimation to analyze a large body of survey data from 964 university students. Although many of the results were consistent with what we hypothesized at the outset, the data indicated three deep-level theoretical paradoxes that contradict or challenge existing cross-cultural cognition models. Each paradox is detailed within the following discussion section.

The first paradox concerns the “Contact Paradox.” Research results indicated that both direct contact experience (DCE) and indirect contact experience (ICE) significantly and positively predicted more positive China–Africa relations cognition (CARC) and closer social distance (SD). This result strongly supports Gordon Allport’s Contact Theory (Allport, 1954) and subsequent Extended Contact Theory (Wright et al., 1997), namely that intergroup contact can promote positive attitudes. However, contrary to theoretical expectations, direct and indirect contact experiences also significantly predicted stronger negative stereotypes about Africa (AS). This indicates that contact, while bringing emotional distances closer, also strengthens negative cognitive schemas, presenting a contradictory state of “emotional approach, cognitive solidification.” The second paradox involves “Interpersonal-Geopolitical Disjuncture.” Data showed that social distance (SD), as a measure of individual intimacy, was not significantly associated with China–Africa relations cognition (CARC), which measures macro-level bilateral relationship cognition, thus hypothesis H3 was not supported. This result challenges a common intuitive assumption that individual-level goodwill can naturally translate into positive evaluations of national relations. It reveals that in university students’ cognition, interpersonal emotional acceptance and geopolitical macro-level judgments may be two mutually independent psychological dimensions. Finally, and most centrally to this study’s findings, is the “Paternalism Paradox.” Completely contrary to the expected direction of hypothesis H4, the study found that negative stereotypes about Africa (AS) not only did not weaken but significantly and positively predicted more positive China–Africa relations cognition (CARC). This surprising result constitutes the greatest theoretical challenge of this study, suggesting an anomalous psychological mechanism: perceptions of Africa as “backward” and “incompetent” actually become the foundation for evaluating China–Africa relations as “good.”

Faced with these three major paradoxes, this discussion proposes and argues for a “Two-Level Cognitive Model” (A Two-Level Model of Perception). This model argues that Chinese university students’ cognitive processes regarding Africa are not singular and linear but are differentiated into two relatively independent yet interconnected levels: one is the emotion-driven “Interpersonal-Affective” level, and the other is the “Geopolitical-Cognitive” level driven by cognitive schemas and ideology. The core argument of this paper is that the above paradoxes, particularly the positive association between negative stereotypes and positive relationship cognition, can be reasonably explained through the concept of “Paternalistic Prejudice” from the Stereotype Content Model (SCM). It is precisely this contradictory stereotype of “high warmth, low competence” that plays a key psychological mediating role at the geopolitical-cognitive level, transforming perceptions of Africa’s “incompetence” into affirmation of the necessity and superiority of China–Africa cooperative relations.

### 5.1 The double-edged sword of contact: confirmation and complication of classic theory

The results of this study are first consistent with contact theory at the macro level. Whether face-to-face direct contact (DCE) or indirect contact through media, education, and other channels (ICE), both were significantly associated with more positive China–Africa relations cognition (CARC) and closer social distance (SD). This finding is highly consistent with conclusions drawn by Pettigrew and Tropp (2006) through meta-analysis of 515 studies, namely that cross-group contact can usually effectively reduce prejudice. In this study, the strong association between direct contact and social distance aligns with Allport (1954) classic assertion that high-quality interaction can break down barriers. Meanwhile, the significant association between indirect contact and China–Africa relations cognition also supports Extended Contact (Wright et al., 1997) and even Parasocial Contact hypotheses (Schiappa et al., 2005), namely that in situations with limited direct interaction opportunities, contact through observation or media can also shape positive intergroup attitudes. This is particularly important for understanding attitude formation among university students who mostly understand the world through media in the globalization era.

A major contribution of the current research lies in illustrating the intricacies of contact theory and its constraints when applied to distinct social settings. Furthermore, the findings indicate that both direct and indirect contact with Africans were associated with higher levels of negative stereotyping about Africa. Therefore, instead of relying on the simplistic assumption that “the more contact there is, the less prejudiced people will be,” future researchers must critically evaluate how the specific context and content of intergroup contact impact attitudes. In particular, the suboptimal nature of direct contact became apparent. While direct contact decreased social distance emotionally, it increased African stereotypes cognitively. This suggests that these interactions failed to satisfy one of Allport (1954) critical requirements for optimal intergroup contact—namely, “equal status.” Additionally, Pettigrew and Tropp (2008, 2011) found that intergroup contact typically decreases prejudice through affective pathways (e.g., decreasing anxiety and increasing feelings of empathy); however, changing cognitive stereotypes is significantly more difficult. The findings from the current study strongly support Pettigrew and Tropp’s conclusions: emotional indicators (SD) improved, while cognitive indicators (AS) worsened. Although Chinese and African university students share an equal identity as classmates at universities in China (Li, 2022), significant structural inequalities exist within their university environments (e.g., socioeconomic status, country-of-origin development level, and scholarship type). These unseen disparities may be unconsciously perceived and reinforced by students during their interactions on campus. Thus, although the campus environment appears to be a neutral setting for intergroup interaction, it may actually function as a site where the central stereotype of “African incompetence” is validated rather than challenged.

Second, the “ideological content” of indirect contact. The positive association between of indirect contact experience (ICE) on negative stereotypes (AS) more directly points to the information content itself that university students encounter. As pointed out in the literature review section of this study, Chinese mainstream media and official narratives, while positive in tone when reporting China–Africa relations, often focus on China’s aid, investment, and leading role, portraying Africa as a passive recipient and object in urgent need of development. This narrative framework constitutes the main content of university students’ Parasocial Contact. Therefore, the more frequently students learn about Africa through these channels (higher ICE scores), the more likely they are to internalize a cognitive schema of “Africa is backward and needs China’s help” (higher AS scores), even though the overall orientation of this information is “China–Africa friendship.” This contact is not neutral information acquisition but more akin to ideological indoctrination that, while promoting cooperation, also systematically reproduces unequal cognitions about competence.

### 5.2 The great divide: the disconnect between interpersonal emotions and geopolitical judgments

A theoretically significant finding of this study is that hypothesis H3 was not supported: no significant path relationship exists between social distance (SD) and China–Africa relations cognition (CARC). The failure of social distance as a classic indicator measuring emotional intimacy and acceptance between individuals to significantly predict macro-level relationship evaluations suggests that in Chinese university students’ psychological world, there exists a profound chasm between interpersonal goodwill and macro-geopolitical judgments. The significance of this “null result” is extraordinary, as it provides empirical annotation from the micro-psychological level for a grand proposition in International Relations (IR) research—the distinction of levels of analysis. Traditional views might take for granted that enhancing friendship between peoples (i.e., reducing social distance) is a prerequisite for improving national relationship cognition. However, this study’s data indicates that a Chinese student can completely be willing to accept an African as a friend, colleague, or even family member (low SD), but their evaluation of the grand topic of “China–Africa relations” follows a completely different cognitive logic.

Political psychology provides a strong explanatory framework for this phenomenon. The literature consistently argues that public views on foreign policy do not exist merely at an emotional level; rather, they are structured by more abstract concepts, including core beliefs, ideological outlooks, and national identity. For instance, Rathbun et al. (2016) demonstrated that individuals’ core belief systems (e.g., universalism vs. conservatism) effectively predict their likelihood of adopting cooperative or confrontational stances in foreign policy. Kertzer et al. (2020) further showed that motivated reasoning shapes how citizens evaluate costly foreign-policy signals, reinforcing the primacy of ideological frameworks over interpersonal affect in geopolitical judgment. Similarly, surveys by Pew Research Center (2019) have consistently shown that when evaluating international relations, the public prioritizes “national interests” over pure international friendship.

Therefore, the “China–Africa Relations Cognition” (CARC) scale measured in this study precisely captures a type of political judgment that transcends individual preferences and carries ideological coloring. Its items involve “mutual benefit and win-win,” “development prospects,” “South–South cooperation model,” etc., all of which are grand narratives about national strategy and international patterns. When students answer these questions, the psychological resources they draw upon are likely not specific emotional experiences from interacting with African classmates, but cognitive frameworks about China’s international role and national interests learned from education, media, and social discourse. The formation of this cognitive framework is a parallel and non-interfering psychological process from their willingness to be neighbors with Africans. This finding profoundly reminds us that when exploring cross-cultural cognition, we must strictly distinguish between attitudes toward “people” and attitudes toward “affairs” (especially inter-state affairs), as there is no taken-for-granted linear transmission relationship between the two.

### 5.3 Solving the core puzzle: paternalistic stereotypes as the foundation of positive relationship cognition

The primary conceptual implication of this investigation is the positive relationship between negative assessments of Africans based upon race/ethnicity (i.e., African Stereotypes, AS) and favorable cognitive appraisals of China’s relationships with Africa (China-Africa Relations Cognition, CARC). This finding reverses the longstanding notion that “negative cognition corresponds to negative evaluation” (H4), necessitating a critical reevaluation of the internal structure of stereotypic beliefs and the role they play within given relational contexts. An explanatory theoretical model for understanding this paradox exists in the Stereotype Content Model developed by Fiske et al. (2002), which posits that group-based stereotypes are fundamentally evaluated along two primary dimensions: warmth and competence. Examination of the items in the AS scale utilized in this investigation clearly illustrates that each item evaluates the degree of perceived incompetence of Africans (e.g., AS3: unskilled labor; AS5: extremely poor economic conditions; AS6: relatively low education levels). Therefore, high scores on the AS scale represent the degree to which an individual has judged another group as having lower competence, and thus should be strictly interpreted as a specific capability assessment rather than a generalized negative attitude toward them based on race/ethnicity.

The Stereotype Content Model provides a theoretical lens for interpreting this pattern. According to Fiske et al. (2002), when an outgroup is perceived as low in competence but high in warmth, the dominant group tends to respond with paternalistic emotions such as sympathy and pity, an ambivalent combination of subjective goodwill and objective disrespect (Cuddy et al., 2007; Glick and Fiske, 2001). Recent experimental work has shown that simply learning about an outgroup’s structural disadvantage can activate competence-undermining paternalism even in the absence of explicit hostility (Adams and Kteily, 2022), and similar paternalistic patterns have been documented in U. S. public support for foreign aid to recipient countries (Baker, 2015). In the present data, the competence dimension was captured by the African Stereotype scale, and a proxy for the warmth dimension was captured by the Cultural Positivity Perception scale through positive evaluations of African cultural appeal, contribution, and inclusiveness. Both dimensions independently and positively predicted relationship cognition.

It is crucial to distinguish between two meanings of the Paternalistic Prejudice (PP) claim that the current results can and cannot support. (a) PP as an explanatory model: The combined influence of a low-competence stereotype and a warm-culture stereotype provides an explanation for why both are able to positively predict favorable relationship cognition. This is an anomaly from the perspective of traditional prejudice-as-negative-evaluation paradigms (Allport, 1954). The current findings are supportive of this model. (b) PP as a statistical pattern in which warmth moderates the effect of competence: This is the formal definition of the Stereotype Content Model (Cuddy et al., 2007), and it predicts a statistically significant AS × CPP interaction. However, in the current sample, this AS × CPP interaction did not predict CARC. Therefore, the findings support (a) but do not support (b). Hence, we frame our contribution as being supportive of the Paternalistic Prejudice interpretation, rather than as a confirmatory test of its configurational structure. Establishing this configurational structure would require longitudinal research that captures temporal precedence, experimental manipulations of perceived competence and warmth, or vignette-based research that isolates the concurrent activation of these stereotypes. Furthermore, the established public discourse on China-Africa friendship and brotherhood (Dent, 2010; Strauss, 2009; Zhang, 2017) supplies a broader narrative context against which the additive co-occurrence of low-competence and high-cultural-positivity perceptions becomes theoretically salient. We emphasize, however, that these contextual interpretations are theoretical rather than empirical: national identity, historical narratives, and system-justifying ideologies (Jost and Banaji, 1994; Pratto et al., 1994) were not directly measured in this study.

How can this “low competence, high warmth” paternalistic stereotype be associated with positive relationship cognition (CARC)? Its internal logic lies in this stereotype providing cognitive foundation and legitimacy for a specific relationship narrative. When China is viewed as a high-competence, high-status actor and Africa is viewed as a low-competence partner needing help, the nature of “China–Africa cooperation” is cognitively defined as a type of assistance and support led by the strong for the weak. This relationship model not only does not suffer because of the other party’s “incompetence” but appears more “necessary,” “noble,” and “productive” because of it. Students’ identification with China–Africa relations as “mutually beneficial and win-win” (CARC1) and “promoting common development” (CARC4) is likely interpreted within this paternalistic framework: China’s “benefit” is strategic influence and resource acquisition, Africa’s “benefit” is obtaining urgently needed development aid and infrastructure construction, with the core driving force of development coming from China. Therefore, stereotypes about Africa’s “incompetence” are not obstacles to positive relationship cognition but become psychological prerequisites for its existence. They make China’s dominant position in the relationship appear natural and benevolent, which corresponds to students giving extremely high positive evaluations of this relationship itself.

### 5.4 Integrative theoretical framework: a two-level model of cross-group and international cognition

To systematically reconcile the seeming contradictions in the findings identified above, this study introduces a “Two-Level Model of Intergroup and International Perception.” The central thesis of this model is that Chinese university students’ cognitive processing of Africa-related information is multi-layered and non-linear, existing on two functional levels (i.e., intergroup perception and international perception), each with its own logic. The Two-Level Model explains how the same contact may have opposite effects, why goodwill at an individual level does not translate to a national level, and how favorable relationship assessments may be based on negative stereotypic beliefs.

#### 5.4.1 Level one: the interpersonal-affective pathway

The primary function of this pathway is to process the individual-to-individual emotional responses of those involved in an interaction, with social distance serving as the key psychological indicator. This pathway is grounded in the principles of classic contact theory. Regardless of whether it occurs through direct or indirect forms of cross-cultural contact, its major goal is to lessen the uncertainty that arises when people are exposed to different cultures and to reduce intergroup anxiety, while enhancing familiarity and empathy (Pettigrew and Tropp, 2008). As such, contact experience (DCE/ICE) has been found to decrease psychological social distance (↓SD), which corresponds to increased acceptance of outgroup members in private settings (e.g., being friends or neighbors). In essence, this is an emotionally driven and depoliticized pathway, focused fundamentally on the question: “Can we be friends?” The significant predictive association between contact experience and social distance found in this study is consistent with this pathway.

#### 5.4.2 Level two: the geopolitical-cognitive pathway

The primary function of this pathway is to process individuals’ perception, judgment, and evaluation of macro-level entities (nations or regions) and their interrelationships. Specifically, it utilizes China-Africa relations cognition (CARC) and African stereotypes (AS) as its core psychological constructs. At this level, the conceptualization of contact undergoes a fundamental shift. Contact is no longer merely an emotional response; rather, it becomes a process through which ideologized knowledge is acquired and validated. In many cases, indirect contact (obtained through media and educational systems) carries embedded, specific narratives concerning national prestige, strength, and international strategy. Drawing on the Stereotype Content Model (SCM), these narratives systematically produce “low competence” stereotypic perceptions regarding Africa. These “low competence” stereotypes are then combined with implicit “high warmth” assumptions to activate paternalistic prejudice. Thus, this prejudice framework provides a set of cognitive shortcuts for evaluating China-Africa relations: a powerful, capable China assisting a friendly, yet backward Africa is inherently perceived as a “positive” relationship. Therefore, within this pathway, the logical sequence is: contact (especially indirect contact) → strengthening of low-competence stereotypes (↑AS) → presence of the paternalistic framework → positive association with relationship cognition (↑CARC).

#### 5.4.3 Model integration and explanatory power

Overall, this conceptual model illustrates that intergroup contact operates simultaneously via two distinct routes with divergent associations. Within the interpersonal-affective route, contact decreases intergroup anxiety, thereby reducing social distance between group members. Conversely, along the geopolitical-cognitive route, biased contact contexts and content inadvertently reinforce negative stereotypes regarding outgroup competence. A profound disconnect exists between these two pathways. While contact may improve individuals’ affective experiences and decrease social distance toward an outgroup, these affective gains do not necessarily translate into enhanced macro-level relationship cognition. This occurs because individuals typically evaluate intergroup relationships through the lens of macro-level national identity and geopolitical frameworks, rather than a micro-level perception of personal goodwill. Furthermore, the model resolves the second paradox identified in the current research: the paternalism paradox. Within the geopolitical-cognitive pathway, the activation of low-competence African stereotypes (AS) is associated with paternalistic prejudice, which paradoxically corresponds to highly positive China-Africa relations cognition (CARC). To clearly illustrate this core argumentative logic, Table 8 synthesizes our main empirical findings, the corresponding paradoxes they reveal, and the theoretical resolutions provided by the two-level model.

### 5.5 Theoretical contributions, broader implications, limitations, and future directions

#### 5.5.1 Theoretical contributions

The empirical evidence from this study contributes to theoretical development in related fields of inquiry. Specifically, it expands and complicates contact theory by demonstrating how individuals exhibit divergent responses to the same contact when situated in contexts of profound structural inequality—namely, that contact can show opposing associations on the affective and cognitive components of prejudice. This supports Paluck et al. (2019) macro-level re-evaluation of contact theory, which noted that while many studies report an overall positive contact effect, there is considerable variability in outcomes depending on environmental differences. The current study extends Paluck et al. (2019) re-evaluation by demonstrating that, even when all parties experience the same societal environment, contact can simultaneously reduce perceived social distance and reinforce competence-undermining stereotypes. This constitutes a paternalism-consistent pattern, aligning with experimental findings by Adams and Kteily (2022) and public foreign-aid preference data observed by Baker (2015). Therefore, future researchers examining contact theory must incorporate two key variables: the structural conditions under which contact occurs (e.g., perceived status hierarchies) and the informational frames surrounding the contact (e.g., media portrayals of helper-helped dynamics).

Second, this study applies of the Stereotype Content Model (SCM) to the field of international relations cognition. Previous research mostly applied SCM to analyze interpersonal or intergroup prejudice (such as gender, race, occupation), while this study suggests that SCM can also serve as a useful analytical framework for understanding public attitudes toward foreign relations. The research illustrates how group-level stereotypes (perceptions of Africans) may be projected onto the level of inter-state interactions, corresponding to contradictory geopolitical emotions (namely paternalistic, sympathetic yet contemptuous positive evaluations). This provides a new theoretical tool derived from social psychology for political psychology and international relations research.

Finally, this study provides micro-psychological foundations for international relations theory. For a long time, the “levels of analysis” problem in international relations theory (such as individual, state, international system) has mostly remained at macro-level discussions. This study, through empirical data, reveals from the level of individual psychological processing the real existence and mutual separation of “interpersonal” and “geopolitical” cognitive levels, providing solid psychological evidence for the distinction between “individual level” and “state level.” Meanwhile, the research also clearly demonstrates how national narratives, ideology, and perceived status hierarchies shape individuals’ views on foreign policy.

#### 5.5.2 Practical and policy implications

The findings of this study have important implications for education, public diplomacy, and the future development of China–Africa relations. For education and public diplomacy, research results warn us that merely promoting more student exchanges or advocating “friendship between peoples” may have limited effects and might even inadvertently strengthen paternalistic arrogance. Educational programs aimed at promoting cross-cultural understanding cannot stop at superficial levels of emotional connection and cultural display but must delve into the cognitive level to explicitly address and deconstruct competence-based stereotypes. Curriculum content should go beyond the singular narrative of “aid and being aided” to introduce more about African countries’ autonomous development, technological innovation, cultural diversity, and agency, cultivating appreciation based on equality and respect.

From a media and communication perspective, the results of this study highlight the tremendous social responsibility borne by media outlets. If the media continue to portray Africa solely as a continent dependent on foreign aid or consistently mired in crises—regardless of whether these crises are framed positively (e.g., benevolent aid) or negatively (e.g., intractable poverty)—they will merely reinforce and reproduce low-competence stereotypes. Media outlets have a duty to provide audiences with a broader, more multifaceted representation of Africa’s vastness and complexity. This includes highlighting continental successes, internal diversity, and Africa’s positive impacts on global affairs. Ultimately, true progress requires a fundamental transformation of public cognitive schemas, moving beyond the mere repackaging of entrenched stereotypes within new emotional frameworks.

The most significant challenge to developing a genuine cooperative relationship is that the deeply ingrained paternalistic attitudes held by the public—particularly among the younger generation—must be directly confronted. Consequently, any long-term strategies or initiatives designed to reshape public perceptions of African countries must prioritize transforming underlying cognitive schemas (e.g., entrenched beliefs regarding outgroup competence), rather than merely enhancing positive affective responses.

#### 5.5.3 Research limitations and future prospects

Despite achieving insightful findings, this study still has some limitations that also point to directions for future research. First, the limitations of cross-sectional data. This study employed a cross-sectional design, revealing strong correlations between variables but unable to establish strict causal relationships. Future research urgently needs to adopt longitudinal designs to track trajectories of students’ stereotype and relationship cognition changes over time after experiencing different types of contact, thereby more accurately testing the causal pathways in the two-level model proposed in this study.

Second, measurement limitations. Although the African Stereotype scale captures the competence dimension of the Stereotype Content Model, the warmth dimension was assessed only through the Cultural Positivity Perception proxy. CPP is an exploratory operationalization, not a validated warmth measure: it taps positive cultural evaluation rather than perceived interpersonal warmth (friendliness, sincerity, trustworthiness; Cuddy et al., 2009; Fiske et al., 2002), and no convergent-validity evidence linking CPP to an established warmth instrument is available within this dataset. The Paternalistic Prejudice interpretation in §5.1 should therefore be read as consistent with, rather than as a confirmatory test of, the warmth-component of the SCM. Developing and validating a Chinese-context SCM scale that directly measures both warmth (e.g., trait items on friendliness, sincerity, and trustworthiness) and competence is a priority for subsequent psychometric work, and would enable a confirmatory configurational test of Paternalistic Prejudice that the present design cannot support. Additionally, the Social Distance scale could benefit from incorporating intensity scores (Mather et al., 2017) in combination with Likert responses to capture more nuanced attitudes.

Third, self-report measures are susceptible to social-desirability bias, especially for sensitive intergroup attitudes. Although the questionnaire used some reverse-coded items and attention checks, future research should consider implicit measures to triangulate explicit attitudes. Fourth, broader theoretical constructs such as national identity, historical narrative, and system-justifying ideology were not directly measured; future research should incorporate such constructs to test the interpretive framework advanced here more rigorously.

Fifth, limitations of sample representativeness. The sample consisted only of mainland Chinese university students, a highly specific subgroup, limiting generalizability to other segments of the Chinese public or to African publics. Our supplementary subgroup analysis by discipline (reported in Table 9) showed that the AS to CARC association is robust across disciplinary subgroups but that the DCE to CARC association is weaker among engineering students, suggesting that disciplinary context may moderate contact effects, a finding that deserves further investigation. Future research should also extend samples across age, occupational, and educational groups to test the generalizability of the two-level cognitive model.

Addressing the aforementioned limitations warrants further research across three distinct methodological domains. Primarily, experimental designs could manipulate media framing—specifically by contrasting emphases on African competence, warmth, or needs—to gage the immediate impact of such narratives on China–Africa relations cognition (CARC). Second, cross-national comparative analyses replicating this framework across diverse African states would determine whether analogous cognitive structures underpin local perceptions of China. Finally, qualitative inquiry, particularly through in-depth interviews with participants exhibiting paternalistic tendencies, would provide deeper insight into the underlying reasoning, emotional responses, and narrative logic that sustain such cognitive patterns.

## 6 Conclusion

This study systematically explored the complex psychological mechanisms influencing Chinese university students’ perceptions of Africa. The research found that university students’ cognitive systems exhibit profound internal tension: cross-cultural contact, while bringing interpersonal emotional distances closer, may also strengthen negative stereotypes about “competence” due to unequal contact contexts and biased media content. More importantly, this study revealed a core paradox: perceptions of Africa’s “incompetence,” through a paternalistic prejudice psychological mechanism, actually become the cognitive foundation supporting positive evaluations of China–Africa relations.

To explain these findings, this study constructed a “Two-Level Cognitive Model” that distinguishes between the “interpersonal-affective” pathway processing personal preferences and the “geopolitical-cognitive” pathway processing macro judgments. This model not only successfully integrates all findings of this study but also provides an integrative theoretical framework combining contact theory, the Stereotype Content Model, and international relations psychology. The conclusions of this study indicate that to construct truly equal and healthy transnational cognition, merely enhancing emotional connections is far from sufficient; the key lies in deconstructing and reshaping those deeply rooted, unequal cognitive schemas about competence and status.

The theoretical contributions of this research are threefold: it complicates contact theory by showing that contact in unequal contexts may simultaneously be associated with positive emotional and negative cognitive outcomes; it extends the Stereotype Content Model to international relations cognition; and it provides micro-psychological evidence for the levels-of-analysis distinction in international relations theory. For practice, the findings suggest that cross-cultural educational programs should address deep-seated competence stereotypes rather than remaining at the level of cultural exchange, and that media representations of Africa should foreground agency and diversity rather than aid dependency. Future research should employ longitudinal and experimental designs to test the causal pathways proposed here, and should extend the sample beyond university students to examine the generalizability of the two-level cognitive model. In sum, by revealing the psychological mechanisms underlying seemingly paradoxical attitudes toward China–Africa relations, this study offers insights relevant to building more equitable perceptions in the context of South–South cooperation.

## Data Availability

The raw data supporting the conclusions of this article will be made available by the authors, without undue reservation.
